# Identification of molecular pattern and prognostic risk model based on ligand-receptor pairs in liver cancer

**DOI:** 10.3389/fimmu.2023.1187108

**Published:** 2023-09-25

**Authors:** Pengbo Hu, Liang Xu, Yongqing Liu, Xiuyuan Zhang, Zhou Li, Yiming Li, Hong Qiu

**Affiliations:** Department of Oncology, Tongji Hospital, Tongji Medical College, Huazhong University of Science and Technology, Wuhan, Hubei, China

**Keywords:** liver cancer, ligand-receptor, molecular pattern, risk model, TME

## Abstract

**Introduction:**

The tumor microenvironment of hepatocellular carcinoma is composed of multiple cells, and the interactive communication between cells drives tumor progression and characterizes the tumor. Communication between cells is mainly achieved through signal transduction between receptor ligands, and the rise of single-cell technology has made it possible to analyze the communication network between cells.

**Methods:**

We applied a train of bioinformatic techniques and in vitro experiments. We analyzed the composition of the microenvironment of liver cancer by combining single-cell sequencing data and transcriptome sequencing data from liver cancer to construct molecular typing and risk models for LRs. Then, we analyzed association of it with prognosis, mutation, KEGG, tumor microenvironment (TME), immune infiltration, tumor mutational burden (TMB) and drug sensitivity in liver cancer. qPCR and was used to identify SLC1A5 expression in LIHC cell lines and CCK8, transwell and cell colony formation were performed to validate the function of SLC1A5. Meanwhile, we also performed polarization of macrophages.

**Results:**

In this experiment, we found that liver cancer tissues are rich in immune and mesenchymal cells, and there is extensive signaling between individual cells, so we constructed molecular typing and risk models for LRs. Combining clinical data revealed significant differences in clinical characteristics, prognosis and mutated genes between the molecular typing of receptor-ligand pairs, as well as in sensitivity to drugs; similarly, there were significant prognostic differences between the risk models. There were also notable differences in activated signaling pathways, infiltrating immune cells and immune subtypes. Subsequently, we used siRNA to knock down SLC1A5 in hepatocellular carcinoma cells and found that cell proliferation, migration and invasion were diminished.

**Conclusions:**

In conclusion, our LRs model may become a marker to guide clinical treatment and prognosis.

## Introduction

HCC ranks as the sixth most prevalent new tumor in the world, but the second most common new death (1). Due to the insidious character of liver cancer, the detection rate of early-stage liver cancer is quite poor, while the highly malignant characteristics of liver cancer frequently result in the identification of liver cancer at an advanced stage. The prognosis for advanced liver cancer is terrible, with a lack of surgical opportunities and a high risk of metastasis within and outside the liver, resulting in worse quality of living and a shorter survival time for patients (2).

The tumor microenvironment, a hothouse of research in recent years, is composed of tumor cells, immune cells and stromal cells that together modulate tumor growth, metastasis, drug resistance and other properties (3). In the last few years, the rapid development of single-cell sequencing has made it possible for scientists to peek into the details of the tumor microenvironment (4). Through the use of gene expression profiles of individual cells, we can identify the specific roles played by different cell types in the tumor microenvironment, refining the function of cells to investigate the microenvironment in a specific target (5).

Cell-to-cell signaling moderates the function and state of the cell. With the continuous enhancement of precision in the research of tumors, receptor-ligand interactions to deliver signals between tumor tissues have attracted the attention of scientists (6). In particular, in the tumor microenvironment, a majority of the interactions between tumor cells, immune cells and stromal cells are mediated by receptors and ligands, of which investigations on PD1 and PD-L1 have made a significant contribution to clinical diagnosis and treatment (7).

Currently, there is no effective method to forecast the prognosis of patients with hepatocellular carcinoma, but receptor-ligand interactions could, up to some extent, anticipate the malignancy of the tumor and thus predict the prognosis of patients. Consequently, we developed a receptor-ligand pairs (LRs) model based on hepatocellular carcinoma to predict the risk of patients and ultimately improve their survival.

## Materials and methods

### Datasets

The single cell sequencing data (GSE146115) was obtained from the GEO database, the gene expression profiling data, clinical data and mutation data from TCGA and ICGC. The ICGC-LIHC sample (231) set was regarded as the external validation set, and the TCGA-LIHC sample (365) set as the training set.

### scRNA-seq data analysis and cell type definition

“Seurat” R package was applied to analyze the expression matrix of single cells, and we screened for cells with the optimal number of genes expressed (50~20000) (8). The mitochondrial genes have been removed from the expression matrix. After controlling for average expression and dispersion relationships, all highly variable genes in single cells were identified. Subsequently, the highly variable genes were used to perform principal component analysis to identify significant principal components as a means of eliminating batch effects based on the “jackstraw” function. Cells were cohorted into 12 different cell types at a resolution of 0.5 using the “FindClusters” function. The “FindAllMarkers” function was applied to discern differentially expressed genes (DEGs). Additionally, a few traditional markers for defining cell subsets were gathered from earlier studies (**Table 1**) and manually annotated in accordance with marker expression.

**Table 1:** The marker genes for the cells.

Cell subgroups	Markers
B cells	CD79A, CD79B
Fiberblast cells	ACTA2
Hepatocytes	CYP2C9, ARG1
Myeloid cells	CD68, CD163
NK cells	NCAM1, GNLY
T cells	CD3D, CD3E

### Cell to cell communication

The various cells of the tumor microenvironment interact with each other to exert tumor promoting or inhibiting effects through activation between various ligands and receptors. Cellular communication was accomplished through “cellphonedb”, a public database containing ligands, receptors, and their interactions, and by annotating the membrane, secreted, and peripheral proteins of each cell subgroup at various time points(9). We conducted the “cellphonedb” to unpack the matrix of cellular communication and we filtered the receptors and ligands that appeared in the expression matrix in TCGA.

### Selection of receptor-ligand pairs

Cellular interactions depend on the simultaneous expression of receptors and ligands, and receptors can only be stimulated to mediate intercellular communication when the number of receptors and ligands is at a parallel elevation. We screened for LRs with receptor-ligand co-expression correlations greater than 0.3 (p < 0.05) and used these LRs for clustering to determine molecular types (10).

### Molecular subtyping calculation

We took the sum of the gene expression of the receptor and ligand as the expression of the LR. We combined the receptor-ligand expression data with the prognostic data to filter out LRs with prognostic significance and then used the R package “ConensusClusterPlus” to generate consensus matrix, “euclidean” was selected as the distance metric for PAM algorithms. A random subset of the TCGA data was selected from the TCGA data, the size of the subset was 80% of the original data set and 500 replications were performed (11). The amount of clusters was varied ranging from 2 to 10 and the most appropriate number of clusters was determined through the calculation of the consensus matrix and the cumulative distribution function (CDF).

### Presumption of drug sensitivity

The R package “pRRophetic” was applied to calculate the sensitivity of different drugs based on expression matrix (12). We determined the appropriate drugs for different classifications of patients based on the sensitivity of the drug, as well as the classification of the different patients.

### Risk model

The R package “glmnet” was conducted to screen for LRs using lasso regression, combined with prognostic data, to build a risk model to predict the risk of patients by classifying them into different risk groups using the median risk score. First, we chose “cv.glmnet” fuction to filter the λ that minimised the discrepancy. Then, based on the value of λ taken at this point, we got to filter the best LRs to build the prognostic model. The risk score was calculated using the formula LR. Score = ∑β(i) ×Exp(i), where i refers to the LR pair, Exp represents the level of LR pair expression and beta is the coefficient of the LR pair in the model. The median value of the training set (TCGA) was selected as the truncation value (13). The R package “survival” was employed to depict survival curves and to compare survival differences between high and low risk groups. The R package “timeROC” to portray the ROC curves of risk scores and traditional prognostic indicators, and to calculate the AUC values to assess the accuracy of the prognosis prediction (14).

### Function enrichment and analysis of mutations and immunity

The “maftools” package was used to visualize the mutation data. We presented the twenty genes with the most significant mutations and compared mutations in patients of different subtypes. We applied “clusterProfiler” to analysis KEGG pathway (15). The ‘hallmark’ gene set collection from the molecular signature database was used for pathway enrichment analysis. Immune cell infiltration assessment was carried out with the “ssGSEA” package (16).

### *In vitro* experimental validation

The cells used in this experiment were obtained from the laboratory of the Department of Oncology, Tongji Hospital, Huazhong University of Science and Technology. LO2, SNU398, Huh7 and HLF were cultured in DMEM medium with 10% fetal bovine serum added. We extracted RNA from the cells, reverse transcribed them into DNA and qPCR detected the expression of SLC1A5 in the cells. Subsequently, SLC1A5 was knocked down in Huh7 and SNU398 cells using siRNA, and CCK8, cell colony formation and transwell were used to detect the proliferation, migration and invasion ability of the cells (17). Meanwhile, we also performed polarization of macrophages. Detailed experimental steps are in the **Supplementary Data Sheet 1**, and all experiments were repeated three times.

### Statistical analysis

Dichotomous variables were tested using the chi-square test, survival analysis was performed using the log-rank test, and comparison between the two groups was performed using the Wilcoxon test. p<0.05 was considered to be statistically different.

## Results

### The single-cell transcriptome landscape of hepatocellular carcinoma

**Figure 1:**
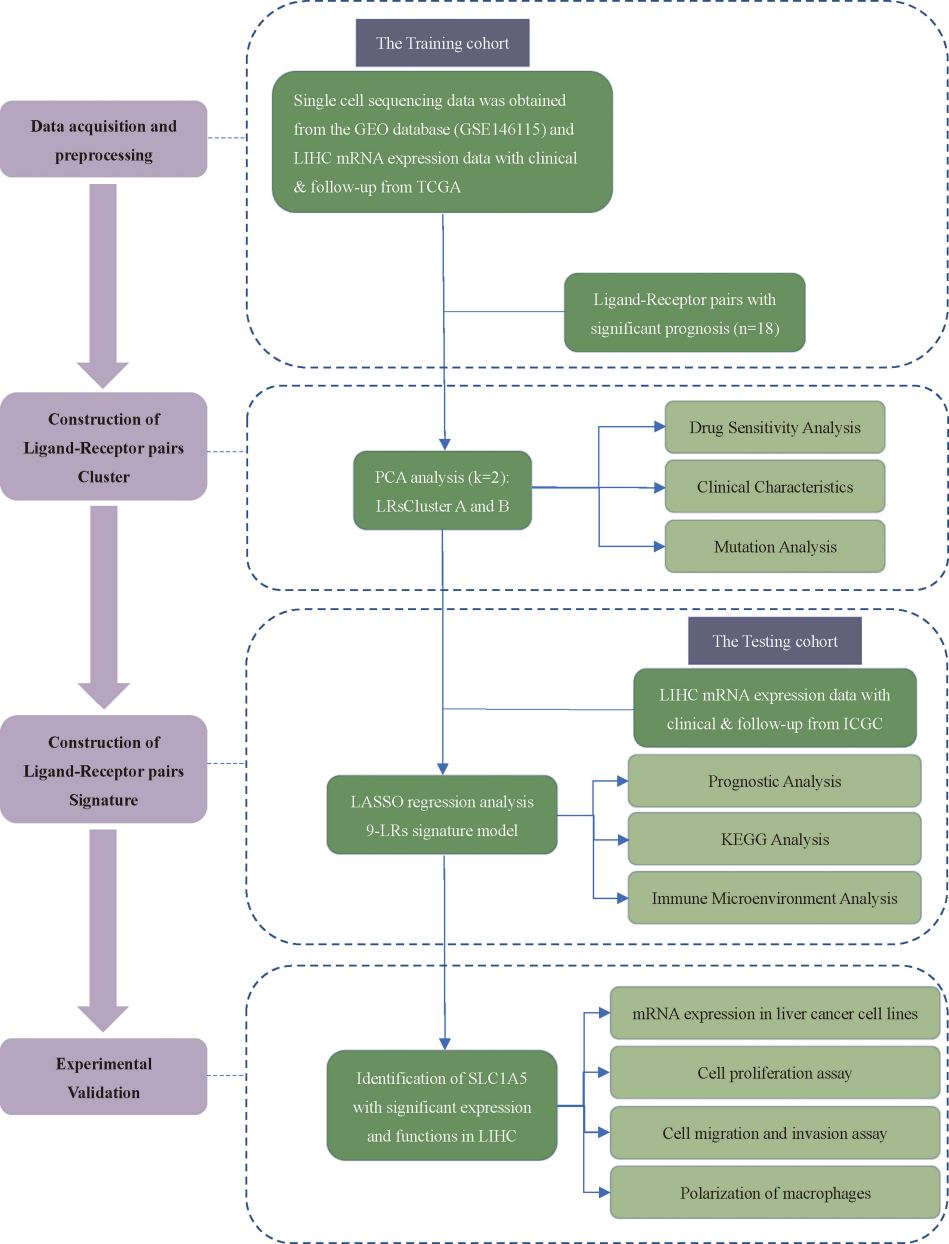
The flowchart of the study design.

**Figure 2:**
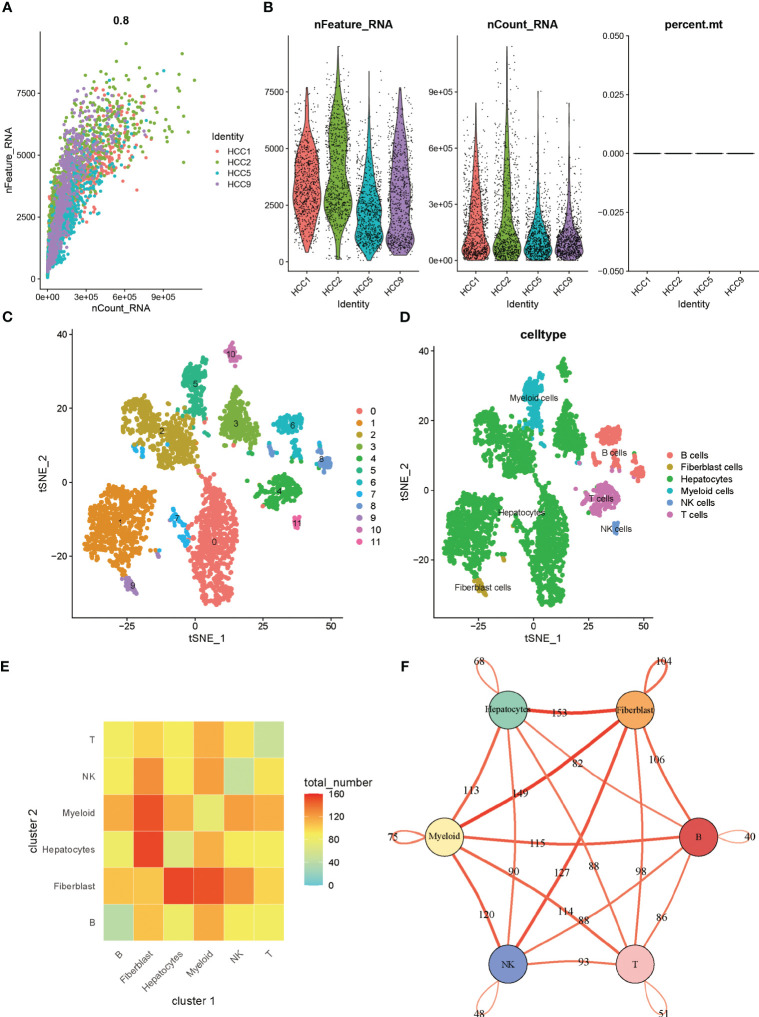
**(A)** Correlation between the number of unique molecular identifiers and mRNA **(B)** Violin plot of features **(C)** tSNE of cell profiles; different color blocks represent related cell clusters. **(D)** tSNE of cell profiles; different color blocks represent related sample sources. **(E)** LR interactions between different cell subsets. **(F)** Network overview for the interaction between different cell subsets.

**Figure 1** showed the overall design and flow chart of this study. Since the gene expression data from single cells excluded mitochondrial genes, we calculated the correlation between the number of unique molecular identifiers and mRNA, which suggests that a significant positive correlation was shown between the number of unique molecular identifiers and mRNA (**Figure 2A**). Gene numbers for the vast majority of cells are between 0 and 8000(**Figure 2B**). After filtering the cells, a total of 3200 cells were included in the subsequent analysis. The differential genes in the various cell types were calculated for a total of 12 cell types after normalizing the expression data and filtering the first 2000 highly variable genes for the subsequent principal component analysis (**Supplementary Figure 1A**, **Figure 2C**). Based on the marker genes in the different cells, we identified the cell types that needed to be labelled. The marker genes for the cells were derived from databases and earlier studies. As a result, six cells were identified, such as B cells, fibroblast cells, hepatocytes, myeloid cells, NK cells, T cells (**Figure 2D**).

### Intercellular communication networks in hepatocellular carcinoma

The tumor microenvironment in solid tumors is composed primarily of stromal cells and immune cells, in addition to the tumor cells themselves. Within the microenvironment, various cells communicate with each other to transmit information to influence tumor progression. When speculating on intercellular communication, we used “cellphonedb” and found that tumor cells contact mainly with fibroblasts and fibroblasts with myeloid cells in liver cancer (**Figure 2E**). In **Figure 2F**, thicker lines indicate more interactions, and the numbers on the lines represent the number of interacting nodes.

### Molecular typing based on ligand-receptor pairs

**Figure 3:**
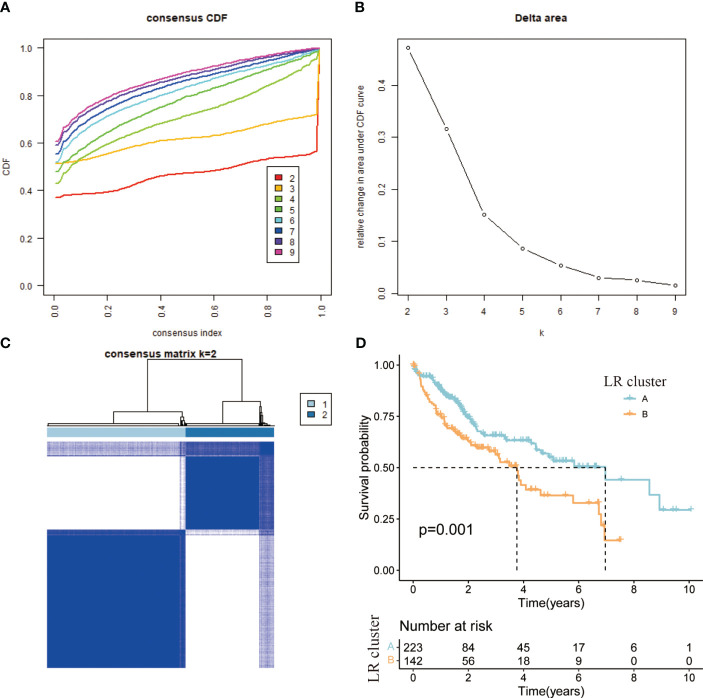
**(A)** CDF curve of samples from TCGA cohort. **(B)** Delta area curve of samples from TCGA cohort. **(C)** TCGA clustering heatmap of samples from TCGA cohort when consensus k = 2. **(D)** Overall survival curves of molecular subtypes based on LR pairs.

In cellular communication, receptor-ligand interactions play an essential role, and through “cellphonedb” simulations we obtained the corresponding LRs. Based on the TCGA expression profile and taking into account the need for synergistic expression of receptors and ligands, we chose LRs with co-expression R-values above 0.3 and P-values less than 0.05. 81 LRs were selected in total (**Supplementary Table 1**). The sum of the expression values of the receptor and the ligand took the place of the LRs’ expression values. Molecular typing analysis was executed on the sample set of TCGA using the “ConsensusClusterPlus”. Based on the CDF value (**Figure 3A**), splitting into two clusters was the preferred candidate when the k value was taken as 2 (**Figures 3B, C**). The ICGC validation dataset was subjected to the same data processing procedure as the TCGA training dataset, and the outcomes were identical. Similar distinctions between the two groups of patients were made based on the LR expression pattern (**Supplementary Figures 2A–C**).

### Comparison of clinical information in different molecular subtypes

**Figure 4:**
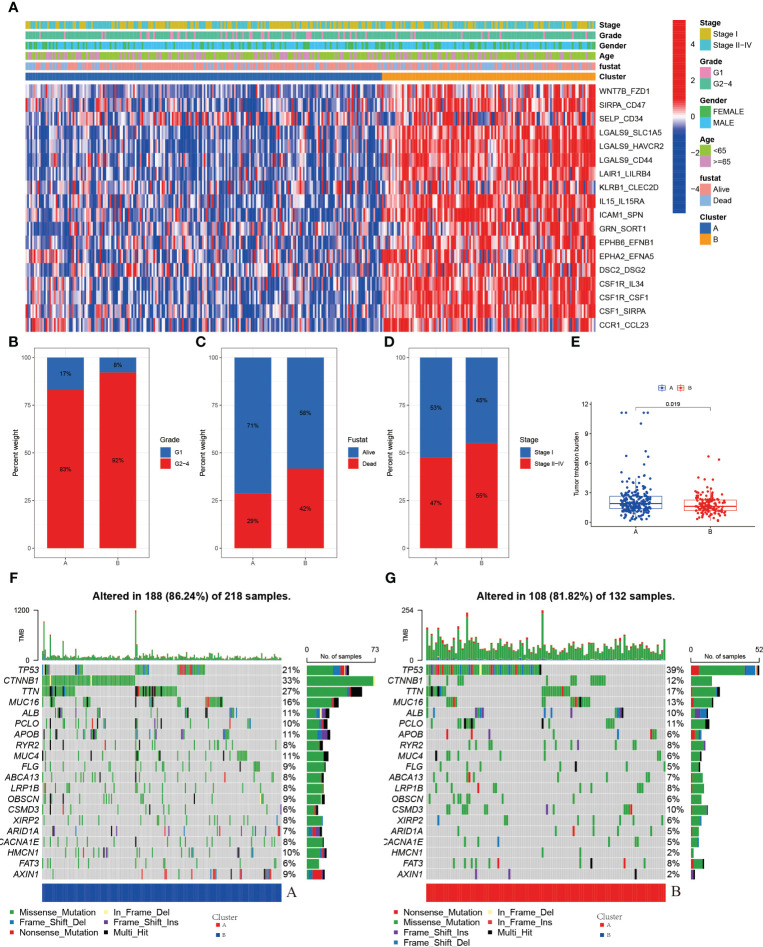
**(A)** Heatmap of expression of LRs in molecular subtypes **(B)** Grade in molecular subtypes **(C)** Fustat in molecular subtypes **(D)** Stage in molecular subtypes **(E)** TMB in molecular subtypes **(F)** Somatic mutation variation analysis in A cluster in the TCGA-LIHC cohort. **(G)** Somatic mutation variation analysis in B cluster in the TCGA-LIHC cohort.

According to the results of the clustering, combined with the prognosis analysis of the patients, we analyzed the survival of the patients in both clusters and the survival curves depicted are shown in the figure. The log-rank test showed that there is a significant difference in survival between the two clusters, with patients in the A subtype having a better prognosis than those in the B subtype in TCGA (**Figure 3D**). The outcomes of the prognostic analyses in the ICGC validation set were agreed with those in the TCGA training set. However, the number of patients in B subtype was too small, perhaps as a result of the limited sample size of patients in ICGC, leading to a P value (0.052) of more than a little over 0.05 (**Supplementary Figure 2D**). Meanwhile, we went on to analyze the gene expression and clinical characteristics of patients in different clusters and found that the expression levels of risk genes were significantly higher in B subtype patients than in A subtype (**Figure 4A**). Regarding the clinical traits, we discovered that patients in the B subtype had greater tumor grade and TNM stage as well as more fatalities than those in the A subtype (**Figures 4B–D**).

### Mutational characteristics of different molecular subtypes

Genetic alterations in tumor cells, mutational inactivation of anti-oncogenes, amplified overexpression of oncogenes, etc., all of which contribute to tumor development, are intimately associated to tumor growth. When we analyzed the mutation data of patients in A and B subtype patients, we found that there were more mutations in the A subtype patients (**Figures 4F, G**). The mutations in the B subtype patients were mainly in the antioncogenes and the types of mutations were mainly missense mutations, such as TP53 (**Figure 4G**).

We then analyzed the tumor mutational burden of patients to predict the efficacy of immunotherapy in patients, and we found that the tumor mutational burden (TMB) was higher in A subtype patients than in B subtype patients (**Figure 4E**), which means that the effectiveness of immunotherapy may be better in A subtype patients than in B subtype patients.

### Drug sensitivity of different molecular subtypes

**Figure 5:**
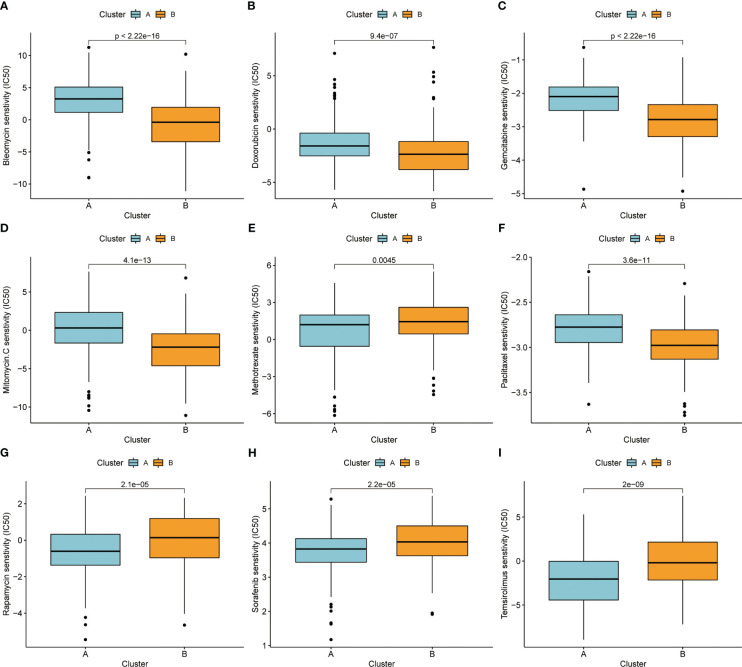
**(A)** Bleomycin. **(B)** Doxorubicin. **(C)** Gemcitabine. **(D)** Mitomycin. **(E)** Methotrexate. **(F)** Paclitaxel. **(G)** Rapamycin. **(H)** Sorafenib. **(I)** Temsirolimus.

We also attempted to see whether there were changes in drug sensitivity amongst the various patient clusters, in addition to examining differences in clinical features and gene expression. Using the R package “pRRophetic” to predict patient sensitivity to chemotherapeutic drugs, we analyzed prominent liver cancer drugs and found that sorafenib, a first-line drug for liver cancer, was more efficacious in B subtype patients. At the same time, we observed that some drugs were more effective in A subtype patients, such as “Bleomycin” (**Figure 5A**),” Doxorubicin” (**Figure 5B**), “Gemcitabine” (**Figure 5C**), “Mitomycin” (**Figure 5D**), “Paclitaxel” (**Figure 5F**), and conversely, some drugs were more targeted in B subtype, such as “Methotrexate” (**Figure 5E**), “Rapamycin” (**Figure 5G**), “Sorafenib” (**Figure 5H**), “Temsirolimus” (**Figure 5I**).

### Establishment of the risk model based on the ligand–receptor pair score

**Figure 6:**
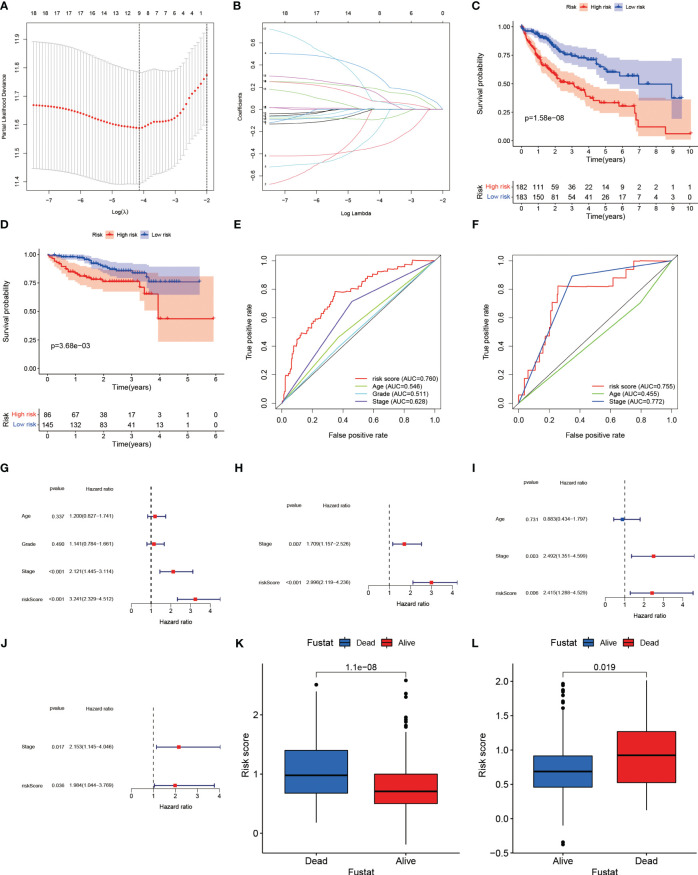
**(A)** Plotting of multinomial deviance versus log(λ). **(B)** LASSO coefficient profiles of the LRs. **(C)** Survival benefit of LR.score in the high and low LR.score groups in the TCGA-LIHC cohort. **(D)** Survival benefit of LR.score in the high and low LR.score groups in the ICGC-LIHC cohort. **(E)** The predictive value of LR.score in patients among the TCGA-LIHC cohort. **(F)** The predictive value of LR.score in patients among the ICGC-LIHC cohort. **(G)** Univariate cox regression analysis of LR.score, age, TNM stage and grade for overall survival (OS) in the TCGA-LIHC cohort. **(H)** Multivariate cox regression analysis of LR.score, and TNM stage for OS in the TCGA-LIHC cohort. **(I)** Univariate cox regression analysis of LR.score, age and TNM stage for overall survival (OS) in the ICGC-LIHC cohort. **(J)** Multivariate cox regression analysis of LR.score, and TNM stage for OS in the ICGC-LIHC cohort. **(K)** Fustat in the high and low LR.score groups in the TCGA-LIHC cohort. **(L)** Fustat in the high and low LR.score groups in the ICGC-LIHC cohort.

Molecular subtypes based on LRs had different clinicopathological characteristics as well as drug sensitivity. We sought to construct a prognostic model using LRs to assess patient risk. A total of 18 LRs associated with prognosis were considered (**Supplementary Figure 1B**), and we used LASSO cox regression to shrink the number of LRs, with optimal results occurring when λ was 0.0159 **Figures 6A, B**). We then used stepwise multivariate regression for optimization, and a total of nine LRs were screened out for model construction (**Supplementary Table 2**). In the training set (TCGA), the median risk score was selected as the cut-off value to classify patients into high and low risk groups, while patients in the validation set (ICGC) were similarly classified into high and low risk groups using the cut-off value in the training set as the boundary.

### Correlation between the risk model and clinical features

Based on the risk groupings, we plotted the survival curves for the training and validation sets separately and used log-rank test to analyze whether there was a difference in survival, and we found that the high-risk group had a worse prognosis in both datasets (**Figures 6C, D**). We also plotted ROC curves based on risk scores and clinicopathological characteristics, and calculated AUC values for different factors separately, and observed that risk scores performed better in predicting patient risk in the training and validation sets (**Figures 6E, F**). In the TCGA training set, the risk model’s sensitivity was 0.784 and its specificity was 0.652; in the ICGC validation set, these values were 0.823 and 0.744, respectively. Both univariate and multivariate analyses showed that the risk score was an independent risk factor to assess patients’ prognosis in TCGA database (**Figures 6G, H**). In the validation set, we similarly found that risk score was also an independent prognostic factor in both univariate and multivariate analyses (**Figures 6I, J**).

The results of the principal components analysis showed that the grouping of high and low risk was able to separate the characteristics of the patients in TCGA (**Supplementary Figure 3A**), and the same phenomenon was observed in the validation set (**Supplementary Figure 3B**). Similarly, we found that the risk scores of surviving patients were significantly lower than those of the deceased in the training set (**Figure 6K**), and the same was witnessed in ICGC (**Figure 6L**). This suggested that the risk score in the prognostic model was highly effective in predicting patient survival, and that patients could be separated according to different risk groupings. In the training set, the proportion of patients who died increased as the risk score increased (**Supplementary Figures 3C, D**). Similarly, we observed the same phenomenon in the training set (**Supplementary Figures 3E, F**).

### Pathway analysis and immune characterization of risk model

**Figure 7:**
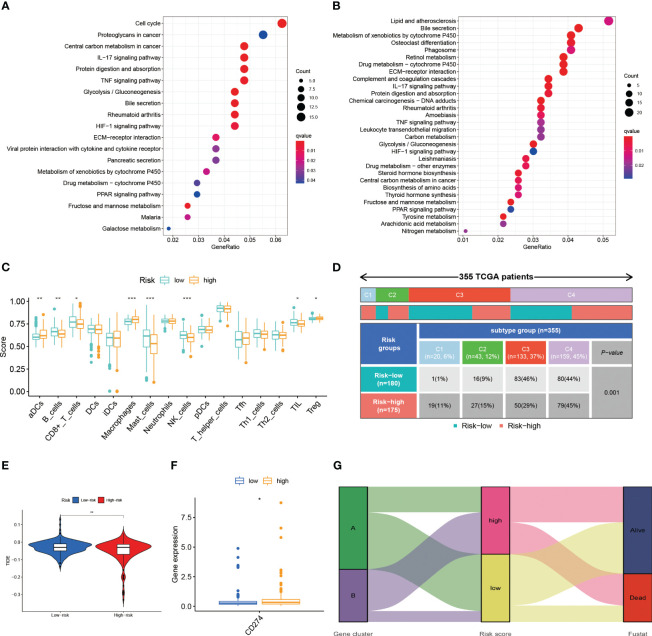
**(A)** The results of the KEGG in TCGA-LIHC cohort. **(B)** The results of the KEGG in ICGC-LIHC cohort. **(C)** Analysis of immune cell scores in the TCGA-LIHC cohort calculated using the ssGSEA algorithms. **(D)** Comparison of immune subtypes in different risk groups **(E)** Correlation of LR.score and TIDE score **(F)** The expression level of CD274 **(G)** Alluvial diagram of the distribution of different gene cluster, risk score and survival outcome subtypes. *p< 0.05; **p< 0.01; ***p< 0.001.

Additionally, we conducted an enrichment analysis of patients in the high-risk and low-risk groups and found that compared to low-risk patients, high-risk patients mainly activate a number of immune-related, metabolism-related and hypoxia-related pathways (**Figures 7A, B**).

We conducted the R package “ssGSEA” to analyze the immune infiltration of patients and we found a decrease in the infiltration of B cells, CD8+ T cells, mast cells, NK cells and, at the same time, an increase in the infiltration of activated DC cells and macrophages in high-risk patients (**Figure 7C**). The reduction of tumor-killing immune cells and addition of tumor-promoting immune cells may be one of the reasons for the worse prognosis in high-risk patients. Combined with the immune phenotyping of the patients, we found that the immune subtypes in the high-risk group were focused on C1 & C2 and in the low-risk group were mostly in C3 (**Figure 7D**).

We compared the levels of PD-L1 expression in the high and low risk groups and discovered that patients in the high-risk group had higher levels of PD-L1 expression (**Figure 7F**). This finding is also consistent with what we found of the ssGSEA above, which revealed that the high-risk group was primarily in an immunosuppressed state. As a result, the high-risk group may be more responsive to immunotherapeutic treatment targeting PD-1/PD-L1.

The TIDE scores are one of the basis for evaluating the current immunotherapy, so we also looked at the TIDE scores of the high and low risk groups. As a result, we discovered that there was a difference in the TIDE scores of the high and low risk groups (**Figure 7E**). Lower TIDE scores in the high-risk group imply that patients in the high-risk group will have a better immunotherapy outcome, which is consistent with the results of the PD-L1 expression level. Patients in subtype A were dominantly in the low-risk group and the state of survival, while the opposite was observed in subtype B patients (**Figure 7G**).

### Functional experiments *in vitro*

**Figure 8:**
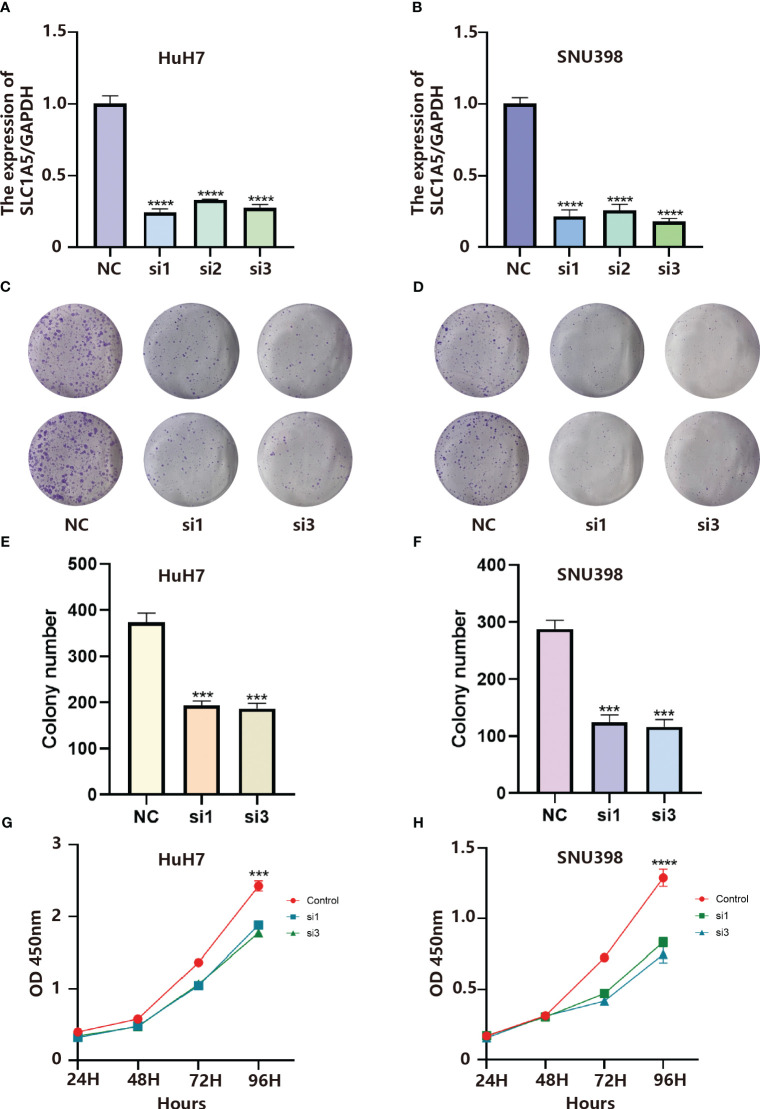
**(A)** Validation of knockdown efficiency by qPCR in HuH7 cells. Results represent mean ± SD. n = 3. ****p < 0.0001; two-tailed t-test. **(B)** Validation of knockdown efficiency by qPCR in SNU398 cells. Results represent mean ± SD. n = 3. ****p < 0.0001; two-tailed t-test. **(C)** SLC1A5 knockdown inhibited colony formation of HuH7 cells. **(D)** SLC1A5 knockdown inhibited colony formation of SNU398 cells. **(E)** The colony number of HuH7 cells. Results represent mean ± SD; n = 3; ***p < 0.001; two-tailed t-test. **(F)** The colony number of SNU398 cells. Results represent mean ± SD; n = 3; ***p < 0.001; two-tailed t-test. **(G)** SLC1A5 siRNA displayed reduced proliferation of HuH7 cells. Results represent mean ± SD; n = 3; ***p < 0.001; two-tailed t-test. **(H)** SLC1A5 siRNA displayed reduced proliferation of SNU398 cells. Results represent mean ± SD; n = 3; ****p < 0.0001; two-tailed t-test.

**Figure 9:**
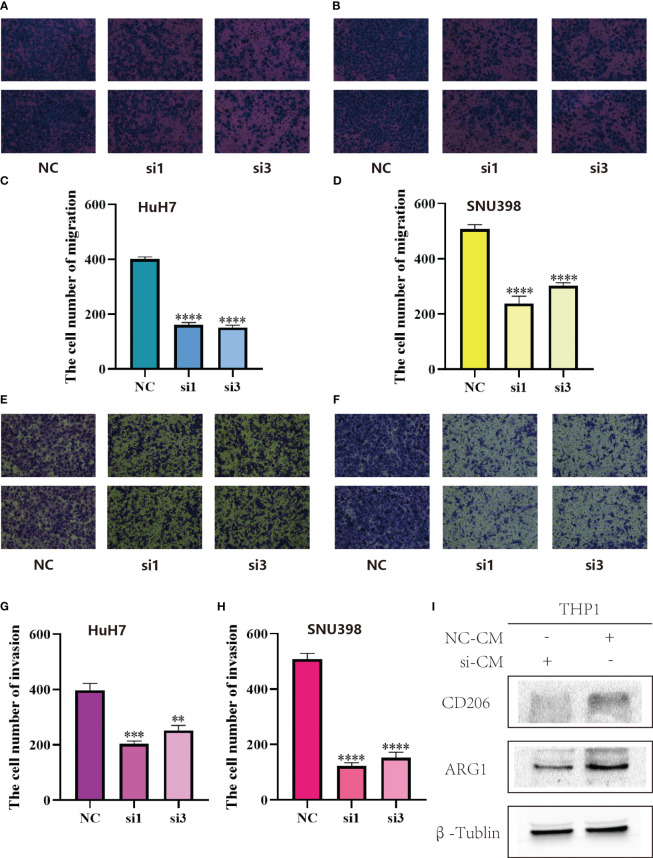
**(A)** SLC1A5 knockdown inhibited migration of HuH7 cells. **(B)** SLC1A5 knockdown inhibited migration of SNU398 cells. **(C)** The cell number of migration of HuH7 cells. Results represent mean ± SD; n = 3; ****p < 0.0001; two-tailed t-test. **(D)** The cell number of migration of SNU398 cells. Results represent mean ± SD; n = 3; ****p < 0.0001; two-tailed t-test. **(E)** SLC1A5 knockdown inhibited invasion of HuH7 cells. **(F)** SLC1A5 knockdown inhibited invasion of SNU398 cells. **(G)** The cell number of invasion of HuH7 cells. Results represent mean ± SD; n = 3; ***p < 0.001; **p < 0.01; two-tailed t-test. **(H)** The cell number of invasion of SNU398 cells. Results represent mean ± SD; n = 3; ****p < 0.0001; two-tailed t-test. **(I)** Relative expression of gene markers of M2 (CD206, ARG1) macrophages by stimulation of different CM in THP1 cell lines.

In the LR model, the coefficient of LGALS9_SLC1A5 was the largest. Considering that SLC1A5 is a receptor, we took a series of experiments to test whether SLC1A5 affects the function of tumor cells. To clarify the expression of SLC1A5, we selected three hepatocellular carcinoma cells and normal hepatic epithelial cells to perform qPCR experiments. We found that the expression level of SLC1A5 was higher in all hepatocellular carcinoma cells than in normal hepatic epithelial cells, with the higher expression level in Huh7 and SNU398 cells (**Supplementary Figure 1C**). We performed the cell proliferation assay (CCK8 and cell colony formation), cell migration and invasion assay (transwell) using siRNA to knock down the expression level of SLC1A5 in Huh7 and SNU398 cells, and observed that the knockdown levels of si1 and si3 were the best among the three siRNAs (**Figures 8A, B**). The results indicated that the proliferation (**Figures 8C–H**), migration (**Figures 9A–D**) and invasion (**Figures 9E–H**) of hepatocellular carcinoma cells were significantly reduced after knockdown of SLC1A5.

After culturing macrophages using conditioned medium from tumor cells, we examined the expression of marker molecules in macrophages. Subsequently, we found that after culturing macrophages using conditioned medium from hepatocellular carcinoma cells with knockdown of SLC1A5, the expression of M2-type macrophage marker molecules was decreased, and the expression levels of CD206 and ARG1 were markedly down-regulated in macrophages (**Figure 9I**). This experimental phenomenon suggests that the expression of SLC1A5 by hepatocellular carcinoma cells may have an effect on the immune microenvironment.

## Discussion

The tumor microenvironment is a burning research topic in the field of oncology. Immune cells are drawn to tumor-associated inflammatory changes, and these immune cells combine with stromal cells in the tumor tissue to form the tumor microenvironment (18). The tumor microenvironment is a double-edged sword in the process of tumor development. Tumor cells will be killed by immune cells to suppress tumor progression. Simultaneously, tumor cells will evade the surveillance of immune cells and gradually remodel the tumor microenvironment so that it promotes tumor metastasis and drug resistance (19).

According to the findings of single cell sequencing, fibroblasts, myeloid cells, B cells, T cells, and NK cells have been found infiltrating liver cancer tissue. The infiltration of T cell kills tumor cells, but recent research has revealed that T cell killing depends on non-depleted T cells and that tumor cells evade the immune system by causing T cell depletion (20, 21). Myeloid cells are predominantly macrophages and tumor cells can induce the conversion of macrophages into tumor-associated macrophages, which are able to interact with other cells either through direct contact or by secreting various effector molecules (22, 23). Tumor-associated fibroblasts contribute to tumor extracellular matrix remodeling, stemness characteristics, angiogenesis and drug resistance (24, 25).

Due to the sophisticated exploration of tumors, the treatment of tumors has evolved from targeting the tumor cells themselves in the early days, directly killing them through radiotherapy or chemotherapy (26), to currently targeting the tumor microenvironment and reversing the cancer-promoting microenvironment to eliminate the tumor cells. Immunotherapy targeting PD-1 on T cells interacting with PD-L1 on tumor cells has been widely used in clinical practice for a considerable period of time (27). Not all patients benefit from them, despite the fact that their efficacy is promising in some tumors (28). Immunotherapies targeting other destinations have also been explored in advance, and we were trying to identify other cellular interactions in the tumor microenvironment that could improve the prognosis of tumor patients. We therefore employed current single cell sequencing to analyze and identify cell types in hepatocellular carcinoma, utilizing software to predict cell-to-cell communication and sought out LRs that were prognostically relevant in hepatocellular carcinoma. Constructing molecular subtypes with different expression patterns of LRs, we observed significant differences in prognosis as well as clinicopathological features of patients in different subtypes, suggesting that we targeted these LRs as possible targets for future clinical therapy. At the same time, we observed that the sensitivity of patients to chemotherapeutic drugs differed between subtypes, which could be the basis for more targeted drug delivery to different patients in the clinical field.

The function of the 18 pairs of LRs for which we constructed molecular typing has been studied in a number of ways. For example, the IL15_IL15RA interaction produces a two-sided effect, with IL-15 promoting the proliferation and maintaining the survival of certain T cells as well as consistently promoting anti-tumor responses and being crucial for controlling tumor growth and metastasis *in vivo*. However, chronic inflammatory stimulation of IL-15 increases tumor growth and metastasis (29). The connection between SELP and CD34 shows that patients gain from the stimulation of the innate immune response to improve anti-tumor immunity, eliminate tumor cells, and hinder the growth of tumors (30). The interaction of the remaining 16 pairs of LRs, in contrast, is what is causing the progression of the tumor, and this interaction allows the tumor microenvironment to change in a way that is pro-cancer, causing M2 type macrophages to transform and inhibiting the activity of NK cells. The main mechanisms of interaction between tumor cells and tumour-associated macrophages are LGALS9-SLC1A5, CCR1_CCL23, CSF1R _CSF1, GRN_SORT1, CSF1_SIRPA, and SIRPA_CD47, which primarily induce macrophage differentiation to M2 type while inhibiting macrophage phagocytosis of tumor cells, cause immune escape, and promote tumor cell proliferation, invasion, and metastasis (31–35). Specifically, LAIR1_LILRB4, EPHB6_EFNB1, and LGALS9_CD44 have immune-suppressive effects. The interactions of LGALS9_CD44 promote the differentiation and maintenance of Treg cells (36). The protein encoded by the EFNB1 gene in EPHB6_EFNB1 is a ligand for type I membrane proteins and Eph-associated receptor tyrosine kinase, and its binding to the ligand primarily exerts suppressive immune effects(37). Tyrosine phosphatases SHP-1 and SHP-2 and/or inositol phosphatase SHIP, which are detrimental to immune activation and promote tumor growth, are recruited by LAIR1_LILRB4 activation (38). WNT7B_FZD1, ICAM1_SPN and EPHA2_EFNA5 are mainly involved in facilitating tumorigenesis and invasive metastasis. Among them, ICAM1_SPN plays an important role in cell-cell interactions, and circulating tumor cells with stem cell properties may be able to use the adhesion protein ICAM1 to promote the formation of circulating tumor clusters that migrate from the body’s primary tumor sites to other organs (39, 40); EPHA2 in EPHA2_EFNA5 induces inhibition of the focal adhesion kinase (FAK), extracellular regulatory protein kinases (ERK) and Akt phosphorylation, thereby regulating motility, viability and proliferation of a variety of malignant cell lines (41).

SLC1A5 is a mitochondrial glutamine transporter and glutamine regulates energy metabolism, signal transduction and redox status in cells (42). Previous studies have indicated that SLC1A5 might affect how immune cells behave and infiltrate tumor microenvironment, which can promote cancer. In breast cancer, SLC1A5 can promote tumor progression (43). Additionally, SLC1A5 accelerates the growth of lung and colorectal malignancies by forcing tumor cells to undergo metabolic reprogramming.

In hepatocellular carcinoma, we observed that the expression level of SLC1A5 was dramatically up-regulated, while the ability of hepatocellular carcinoma cells to proliferate was significantly down-regulated after SLC1A5 expression was silenced using siRNA. Additionally, hepatocellular carcinoma cells’ capacity for migration was noticeably suppressed. The results suggested that the malignancy of hepatocellular carcinoma cells increased when the ligand agonized SLC1A5, and targeting SLC1A5 could alleviate tumor progression in hepatocellular carcinoma patients in the future (44, 45).

In the prognostic model we constructed, there were disparities in immune cell infiltration between the high and low risk groups, with fewer anti-cancerous immune cells and more pro-cancerous immune cells in the high-risk group, which contributed to the worse prognosis in the high-risk group. Meanwhile, patients in the low-risk group for hepatocellular carcinoma were primarily clustered in the C3 immune subtype, and the C3 type was inflammatory, in line with the results of the previous analysis (46). Most of the patients with the molecular subtype A belonged to the low-risk category. Patients in subtype A had a higher tumor mutational burden, and in relation to the previous analysis, we hypothesized that immunotherapy was more effective in subtype A patients. Moreover, both in the validation and training sets, the high-risk group focused on activating tumor metabolism (47), cell cycle, hypoxia, and immune-related pathways compared to the low-risk group. Activation of these pathways tended to make tumor worse, implying a poorer prognosis.

The functions of the 9 pairs of LRs used to construct the prognostic models also vary, among which LGALS9_HAVCR acting primarily as a promoter of lymphocyte activation. As a co-stimulatory molecule during lymphocyte activation, HAVCR enhances the anti-tumor effects of lymphocyte and induces changes in the tumor microenvironment, leading to efficient anti-tumor immunity (48). KLRB1 in KLRB1_CLEC2D is the gene encoding human CD161. KLRB1 gene inactivation or antibody-mediated KLRB1 blockade enhances T cell-mediated glioma cell killing *in vitro*, and the CD161_CLEC2D pathway defines a potential target for immunotherapy of glioma and other human cancers (49). Therefore, increased expression of KLRB1_CLEC2D and LGALS9_HAVCR suggests that patients may have better efficacy of immunotherapy.

Considering the limitations piece, there were three flaws in this paper. Firstly, the studies in the article were retrospective studies that underwent analysis after data collection, the actual clinical value of which had not yet been ascertained in genuine clinical practice. Additionally, the article contained just a limited amount of data, subsequently a larger sample size is supposed to be incorporated into the study. Moreover, the data in the article were partially biased.

## Conclusion

In conclusion, we have analyzed the interactions between cells in hepatocellular carcinoma, thereby establishing molecular subtypes of cellular communication as well as a prognostic model. The expression pattern of LRs may be able to predict the effect of chemotherapy and immunotherapy in patients with hepatocellular carcinoma and to forecast the prognosis of patients. Our findings highlight the clinical implications of LRs and provide a basis for subsequent clinical translation.

## Data availability statement

Publicly available datasets were analyzed in this study. The datasets can be found here: GEO database (HYPERLINK "https://www.ncbi.nlm.nih.gov/geo/"Home - GEO - NCBI (nih.gov)), ICGC database (HYPERLINK "https://dcc.icgc.org/"Welcome | ICGC Data Portal) and TCGA database (HYPERLINK "https://portal.gdc.cancer.gov/"GDC (cancer.gov)). Other datasets presented in this study can be found in online repositories. The names of the repository/repositories and accession number(s) can be found in the article/**Supplementary Material**.

## Author contributions

All authors contributed to data analysis, drafting or revising the article, have agreed on the journal to which the article will be submitted, gave final approval of the version to be published, and agree to be accountable for all aspects of the work. All authors contributed to the article and approved the submitted version.

## Supplementary material

Supplementary Figure 1
**(A)** Heatmap showing marker genes for each cluster. **(B)** The HR values of LRs. **(C)** Quantitative polymerase chain reaction (qPCR) displayed upregulation of SLC1A5 in liver cancer cells compared to normal cell line (LO2). Results represent mean ± SD; n = 3. ****p < 0.0001; ***p < 0.001; two-tailed t-test.

Supplementary Figure 2
**(A)** CDF curve of samples from ICGC cohort. **(B)** Delta area curve of samples from ICGC cohort. **(C)** ICGC clustering heatmap of samples from ICGC cohort when consensus k = 2. **(D)** Overall survival curves of molecular subtypes based on LR pairs.

Supplementary Figure 3
**(A)** The results of the PCA in TCGA-LIHC cohort. **(B)** The results of the PCA in ICGC-LIHC cohort. **(C)** Distribution of risk score (high or low) in TCGA. **(D)** Distribution of status (dead or alive) in TCGA. **(E)** Distribution of risk score (high or low) in ICGC. **(F)** Distribution of status (dead or alive) in ICGC.

Supplementary Table 1 The selected LRs with R-values above 0.3 and P-values less than 0.05.

Supplementary Table 2 LRs associated with prognosis.

Supplementary Data Sheet 1 Detailed procedures of cell transfection, qPCR, CCK8, transwell, colony formation, western blotting, polarization of macrophages, collection of conditioned media and LR.score.
