# Supplementary figures and images for: Identification of molecular pattern and prognostic risk model based on ligand-receptor pairs in liver cancer
Source: Front Immunol. 2023 Sep 25;14:1187108. doi: 10.3389/fimmu.2023.1187108 (PMC10560727; doi:10.3389/fimmu.2023.1187108)

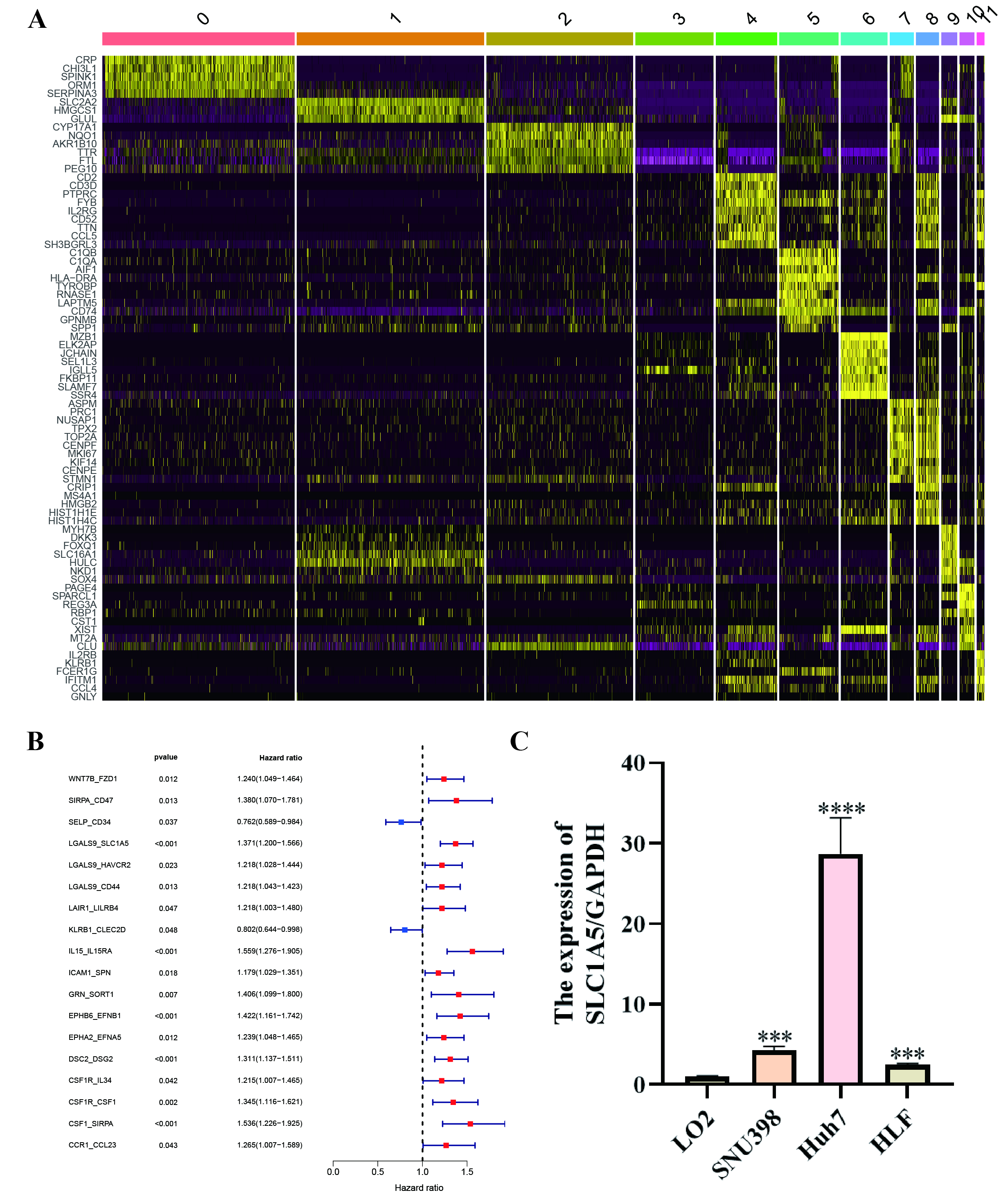

Supplement: Supplementary Figure 1 — (A) Heatmap showing marker genes for each cluster. (B) The HR values of LRs. (C) Quantitative polymerase chain reaction (qPCR) displayed upregulation of SLC1A5 in liver cancer cells compared to normal cell line (LO2). Results represent mean ± SD; n = 3. ****p < 0.0001; ***p < 0.001; two-tailed t-test. [file Image_1.tif]

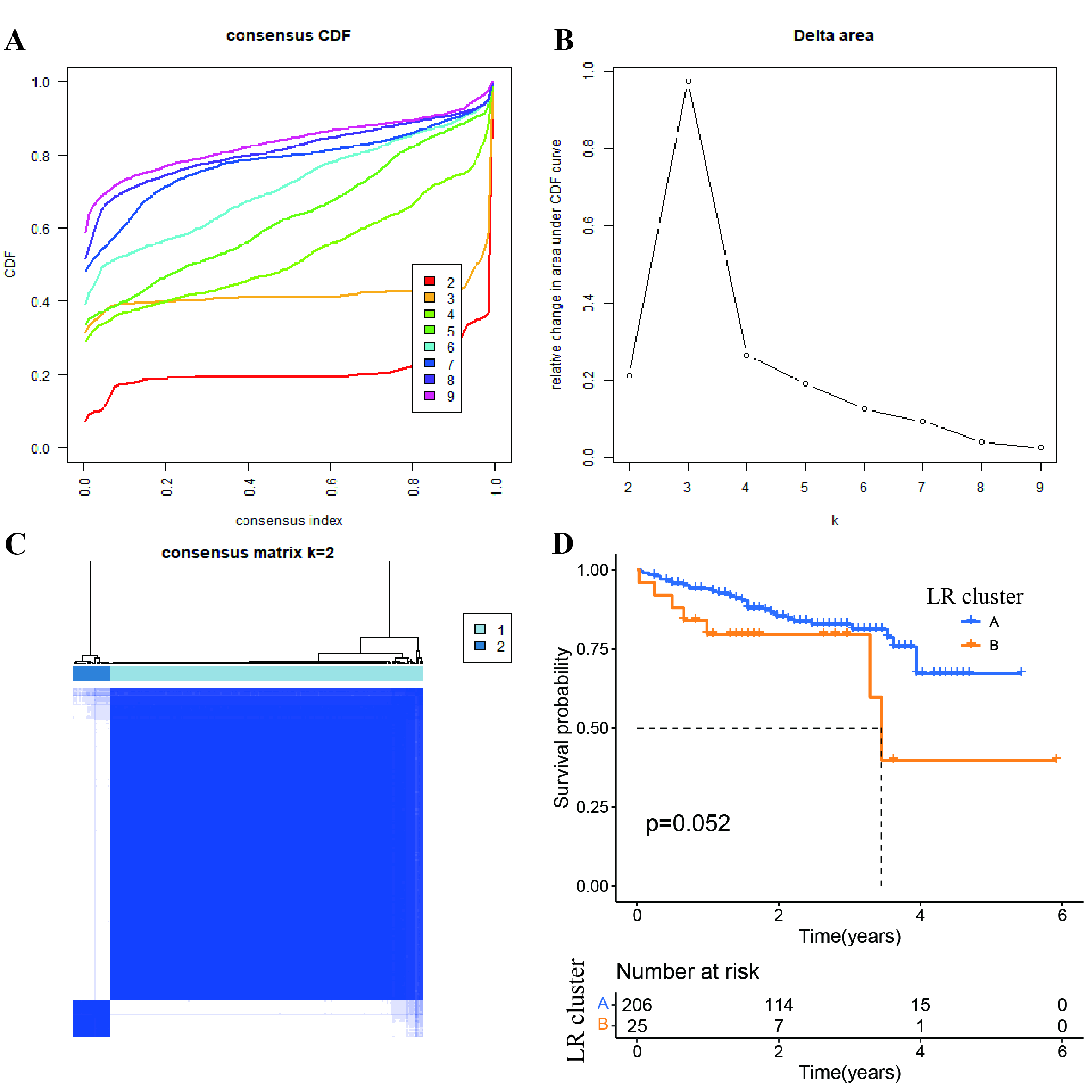

Supplement: Supplementary Figure 2 — (A) CDF curve of samples from ICGC cohort. (B) Delta area curve of samples from ICGC cohort. (C) ICGC clustering heatmap of samples from ICGC cohort when consensus k = 2. (D) Overall survival curves of molecular subtypes based on LR pairs. [file Image_2.tif]

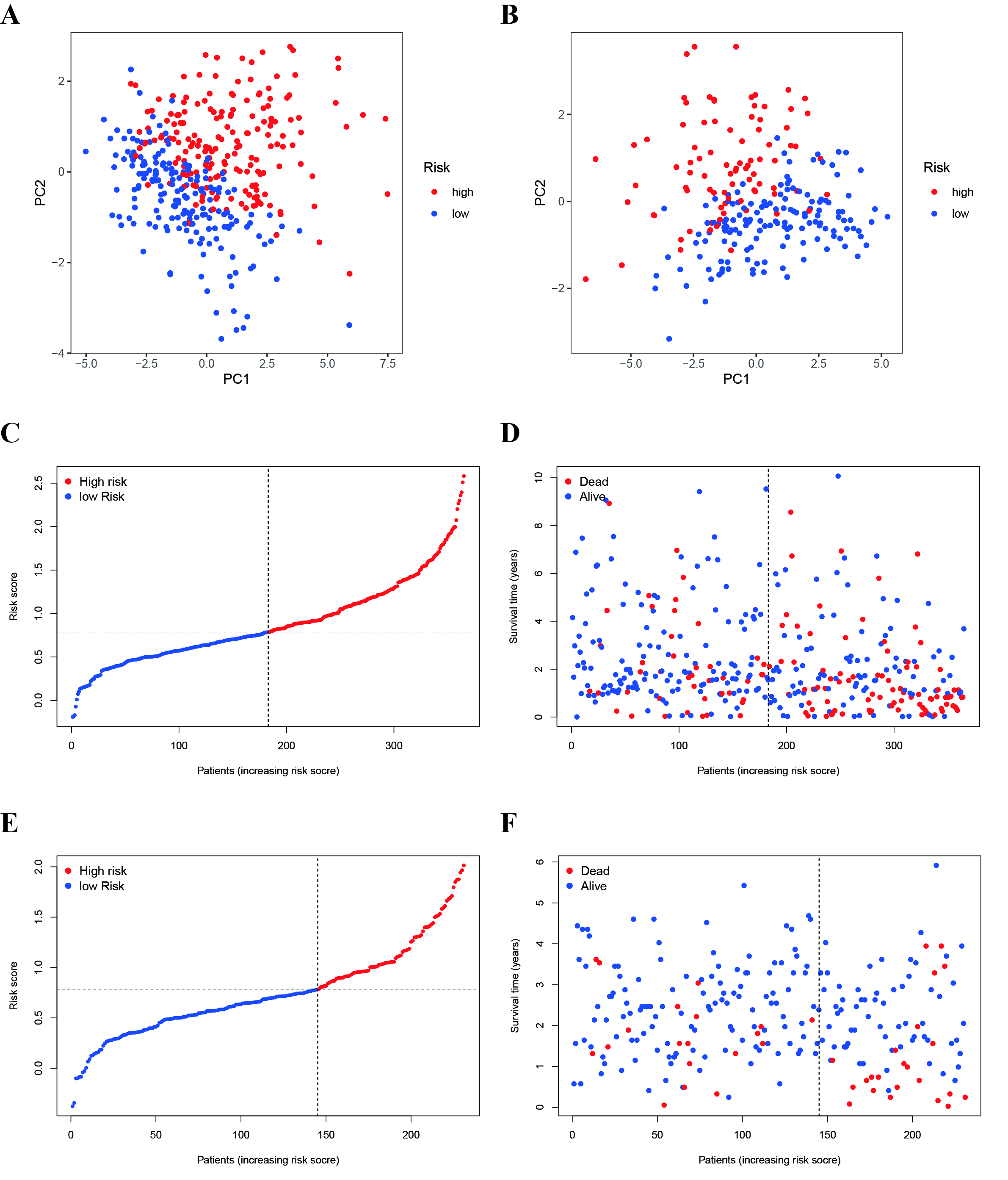

Supplement: Supplementary Figure 3 — (A) The results of the PCA in TCGA-LIHC cohort. (B) The results of the PCA in ICGC-LIHC cohort. (C) Distribution of risk score (high or low) in TCGA. (D) Distribution of status (dead or alive) in TCGA. (E) Distribution of risk score (high or low) in ICGC. (F) Distribution of status (dead or alive) in ICGC. [file Image_3.tif]
