# Supplementary material for: Identification of molecular pattern and prognostic risk model based on ligand-receptor pairs in liver cancer
Source: Front Immunol. 2023 Sep 25;14:1187108. doi: 10.3389/fimmu.2023.1187108 (PMC10560727; doi:10.3389/fimmu.2023.1187108)
Supplement: Supplementary Data Sheet 1 — Detailed procedures of cell transfection, qPCR, CCK8, transwell, colony formation, western blotting, polarization of macrophages, collection of conditioned media and LR.score. [file DataSheet_1.docx]

**Cell transfection**

The tumor cells were extracted and seeded in a six-well plate for one day, and when the cell density reached about 70%, the siRNA (OBiO, China) and lipo3000 (Invitrogen, USA) were diluted separately using Opti-MEM medium (Invitrogen, USA), mixed and left to stand for 20 minutes, then added to the six-well plate. 6-8 hours later, the solution was changed and subsequently the tumor cells were spread over the entire six-well plate.

**Real-Time Quantitative PCR**

After the cells had been spread throughout the six-well plate, 1 ml of TRIzol (TaKaRa, Japan) was added and the RNA was extracted according to protocol. Reverse transcription was carried out according to the requirements of the reverse transcription reagent and instrument (Thermo, USA). The relative expression of genes was finally calculated according to the SYBR Green PCR kit (Thermo, USA) and the 2^−ΔΔCt^ method.

All primers were listed as follows: SLC1A5, Forward: GGTTACTCCTCAAAACCCCCA, Reverse: GTGACCTGCTCCCTGAGACA. GAPDH, Forward: TGCACCACCAACTGCTTAGC, and Reverse: GCGCCCAATACGACCAAATC.

**CCK8**

Cells (1000 per well) were seeded in advance in ninety-six well plates and after overnight, the medium was replaced with 100 µl of serum-free medium with 10% CCK8 (MCE, USA), followed by incubation for 4 hours in a 37 degree incubator. The OD values at 450 nm were detected through microplate reader (BioTek, USA). These steps were repeated at 24, 48, 72 and 96 h.

**Transwell**

For transwell migration assay, 4 × 104 cells were seeded on the upper transwell chambers in 200 μl serum-free culture medium, and 600 μl medium containing 20% FBS was added to the lower chambers. After 24 h incubation, the cells that migrated through membranes were fixed with methanol, stained with 1% crystal violet and counted under light microscope (200×).

**Colony formation**

Cells were seeded in triplicate into 6-well plates (500 cells/well) and incubated for 14 days, fixed with paraformaldehyde for 15 min and stained with 0.1% crystal violet (Servicebio, Wuhan, China) for 20 min. Colonies including outnumbered 50 cells were counted under a microscope.

**Western blotting**

All cells were lysed in radioimmunoprecipitation assay lysis buffer with 1% phosphatase inhibitor Cocktail Ⅱ (100 × in ddH2O) and 1% protease inhibitor cocktail (EDTA-Free, 100 × in DMSO) (MedChemExpress). Measure protein concentration with BCA protein assay kit (Promoter, Wuhan, China). After mixing with 5 × sodium dodecyl sulfate (SDS) loading buffer in a 4:1 volume and boiling for 5 minutes, equal amounts of different proteins were separated by 10% SDS-polyacrylamide gel electrophoresis and wet-transferred to poly (vinylidene fluoride) (PVDF) membrane (Millipore, Billerica, MA). TBST containing 5% non-fat milk powder was used to block the membranes at room temperature for 1 h and then primary antibody was incubated overnight at 4 ℃. Primary antibodies included anti-β-Tublin (1:10000, 10094-1-AP, Proteintech), anti-CD206 (1:1000, #24595, Cell Signaling Technology), and anti-Arginase-1 (1:1000, #93668, Cell Signaling Technology). Then washed membranes in TBST for 10 min three times and incubated with HRP goat anti-mouse or anti-rabbit (Promoter, Wuhan, China) for 1 h at room temperature. At last, the membranes were washed and detected with an ECL HRP Substrate kit (#K22020, Abbkine, Inc.).

**Polarization of macrophages and collection of conditioned media**

THP-1 cells were induced to mature macrophages with 100ng/ml phorbol 12-myristate 13-acetate (PMA; HY-18739, MCE) for 48 h. The cells were cultivated in 6-well plates at a density of 5x10^5 cells/well. To prepare conditioned media (CM), HuH7-NC and HuH7-si1 cells were cultured in a standard medium in 6-well plates. The next day, the cells were washed and cultured with DMEM deprived of FBS for 48 h. To remove dead cells and cell debris, the received media were centrifuged for 10 min at 1500 rpm and 4°C. Then supernatants were concentrated via 10 kDa ultrafiltration centrifuge tubes (Millipore, USA) to use as a conditioned media.

**LR.score**

The formula for the LR.score is as follows: The LR.score = (-0.0921 × SELP_CD34 exp.) + (0.3145 × LGALS9_SLC1A5 exp.) + (-0.2087 × LGALS9_HAVCR2 exp.) + (-0.3386 × KLRB1_CLEC2D exp.) + (0.1876 × IL15_IL15RA exp.) + (0.0734 × DSC2_DSG2 exp.) + (0.0014 × CSF1R_CSF1 exp.) + (0.0767 × CSF1_SIRPA exp.) + (0.1532 × CCR1_CCL23 exp.)
